# An Ensembled Framework for Human Breast Cancer Survivability Prediction Using Deep Learning

**DOI:** 10.3390/diagnostics13101688

**Published:** 2023-05-10

**Authors:** Ehzaz Mustafa, Ehtisham Khan Jadoon, Sardar Khaliq-uz-Zaman, Mohammad Ali Humayun, Mohammed Maray

**Affiliations:** 1 Department of Computer Science, Comsats University Islamabad, Abbottabad Campus, Islamabad 22060, Pakistan; ehtishamkhan2021@gmail.com (E.K.J.); skhaleeq@cuiatd.edu.pk (S.K.-u.-Z.); 2 Department of Computer Science, Information Technology University of the Punjab, Lahore 54590, Pakistan; ali.humayun@itu.edu.pk; 3 Department of Information Systems, King Khalid University, Abha 62529, Saudi Arabia; mmarey@kku.edu.sa

**Keywords:** deep learning, breast cancer, prognosis, diagnostics, DNN, CNN, RNN, LSTM

## Abstract

Breast cancer is categorized as an aggressive disease, and it is one of the leading causes of death. Accurate survival predictions for both long-term and short-term survivors, when delivered on time, can help physicians make effective treatment decisions for their patients. Therefore, there is a dire need to design an efficient and rapid computational model for breast cancer prognosis. In this study, we propose an ensemble model for breast cancer survivability prediction (EBCSP) that utilizes multi-modal data and stacks the output of multiple neural networks. Specifically, we design a convolutional neural network (CNN) for clinical modalities, a deep neural network (DNN) for copy number variations (CNV), and a long short-term memory (LSTM) architecture for gene expression modalities to effectively handle multi-dimensional data. The independent models’ results are then used for binary classification (long term > 5 years and short term < 5 years) based on survivability using the random forest method. The EBCSP model’s successful application outperforms models that utilize a single data modality for prediction and existing benchmarks.

## 1. Introduction

**Figure 1:**
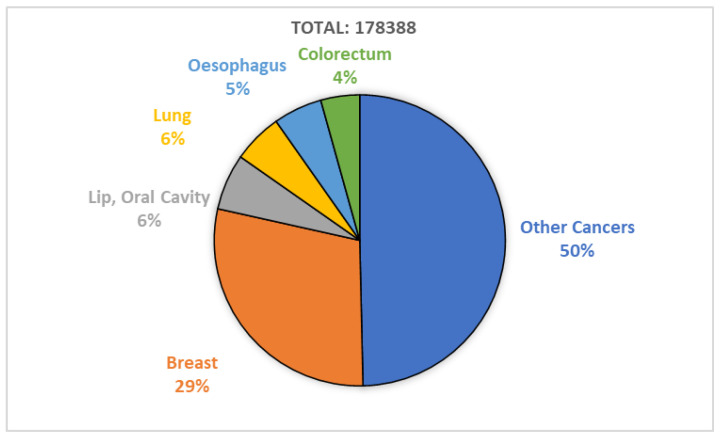
Breast cancer diagnosed in 2020; report by WHO.

The human body is made up of approximately 30 trillion cells. Cancer originates from abnormal cell growth, resulting in the formation of a primary tumor [1]. Breast cancer predominantly affects women due to the excessive growth of breast cells. It is a highly invasive tumor and a leading cause of female fatalities [2]. In Pakistan, breast cancer is prevalent, with one out of every nine women at risk of the disease, and it has the highest cancer-related mortality rate [3]. According to the World Health Organization’s 2020 report, breast cancer is a significant cause of accidental death in women, with 8.2% of the Pakistani population dying from cancer. Figure 1 shows that breast cancer is the most commonly diagnosed cancer type, with a 28.7% diagnosis rate in 2020 [4]. Breast cancer has two types, including malignant and benign. Benign (non-invasive) cancer is a type of cancer that does not affect other organs. On the other hand, malignant (invasive) cancer spreads to neighboring tissues, making invasive cancer prognosis challenging due to varying clinical outcomes [5]. Thus, early and precise diagnosis and prognosis are crucial for timely decision making by physicians to improve patients’ survivability. Survivability can be categorized as short-term (<5 years) or long-term (>5 years). Prognostications aid physicians who work with short-term survivable patients with a multi-featured disease [6].

During the past few decades, the rapid growth of machine learning and deep learning techniques with high throughput has provided deep insights into micro-arrays, gene expression, and clinical data. Machine learning aids in efficiently diagnosing and treating life-threatening diseases, whereas deep learning helps extract highly informative features for disease prediction and prognosis [7]. There are many sources of informative data for breast cancer prognosis, such as genetic data (gene expression and copy number variations) and clinical data (age, pregnancy timing, lifestyle factors, early menstruation, late menopause, etc.). Ensemble learning integrates the prediction of multiple neural networks to reduce the variability in prediction and decrease generalization errors [8]. Integrating the available multi-dimensional data can lead to an efficient breast cancer prognosis [9]. In recent years, researchers have proposed multiple homogeneous prognosis methods based on neural network models using ensemble techniques.

This works relies on developing heterogeneous models based on a prognostic model with stacking. The primary concern is to ensure the heterogeneity of the models for multi-modal data concerning the nature of the data. Different from prior works, we aim to design an LSTM module for the feature extraction of gene expression data. The proposed framework operates through three stages, specifically feature extraction, stacking, and classification. The following are the novel contributions of the proposed framework:

- To obtain highly informative features, we implement an individual neural network for each data modality, including clinical data, gene expression data (Gene-Exp), and copy number variations (CNV).
- Specifically, we design a CNN for clinical data, a DNN for CNV, and an LSTM architecture for the gene expression modality. The output features generated by the individual neural networks are stacked to validate the generalization of the result.
- To confirm that integrating multi-modal data improves the prognostic power, we compare this technique with existing benchmarks. The results validate the effectiveness of the EBCSP in terms of accuracy.

## 2. Related Work

Machine learning has become a popular approach for predicting various diseases, including those affecting the lungs, breast, and oral cavities. In one study, the authors [10] presented an overview of machine learning in primary lung cancer, highlighting both the strengths and weaknesses of these methods. Another study [11] explored the performance of different machine learning models, such as logistic regression, random forest, K-nearest neighbors, and decision trees. The results showed that logistic regression outperformed the other models. In a separate study, Zhou et al. [12] used the Bayesian approach to relate gene expression with class labels, utilizing the Markov chain Monte Carlo (MCMC) method to identify essential genes. The researchers implemented the Gibbs sampler and MCMC using the posterior distribution of the selected genes and validated their proposed method using large micro-array datasets.

In [13], another overview was presented, which showcased recent advancements in radiomics utilizing deep learning. The authors investigated the effectiveness of deep learning in developing predictive and prognostic models. In [14], the authors integrated a CNN and RNN to predict colorectal cancer using tumor tissue samples. The study aimed to directly use patient outcomes, eliminating the need for intermediate tissue classification. Furthermore, a comprehensive analysis of deep learning-based models, including Cox-net, Deep Surv, and Auto-Encoder with Cox regression networks (AECOX), was performed on the TCGA cancers dataset [15]. The study’s findings emphasized the relationship between survival learnability on a pan-cancer level and patient characteristics.

Deep learning with ensemble models is gaining popularity for predicting various types of cancer. In [16], the authors presented a pre-processing data approach that involved feature selection and aggregating random under-sampling. The results showed that an ensemble classifier with a BiLSTM or BiGRU model and a CNN model achieved the best classification performance, with accuracy and F1-score ranging between 91% and 96% for different types of heart disease. This proposed framework can potentially lead to highly accurate models suitable for real clinical data and diagnostic use.

In [17], the authors proposed a novel methodology for classifying human cancer diseases based on gene expression profiles. The proposed system combined Information Gain and Standard Genetic Algorithm for feature selection and reduction, respectively, and Genetic Programming for cancer type classification. The methodology was evaluated on seven cancer datasets and compared with other machine learning approaches. Using a Genetic Algorithm improved the classification performance compared to other classifiers. In [18], the researchers proposed a deep learning-based approach for detecting red lesions in fundus images, which is relevant for detecting early signs of diabetic retinopathy. The method combined deeply learned features with manually engineered features and used a random forest classifier to identify true lesion candidates. The proposed approach showed improved results and reported the highest performance on the DIARETDB1 and e-ophtha datasets.

Breast cancer prognosis is a critical need, alongside the prognosis of various other cancers. In pursuit of this objective, an ensemble approach that incorporates multiple machine learning models was investigated in [19]. The authors considered five different classification models and applied gene expression analysis to these models to obtain informative gene data. The proposed model was validated in lung cancer, stomach adenocarcinoma, and invasive carcinoma samples. The results confirmed the effectiveness of the proposed ensemble model. Another ensemble model was proposed in [20]. Here, the authors considered three classifiers, including support vector machines, logistics regression, and stochastic gradient descent optimization, for breast tumor classification. The proposed complex voting mechanism provided better results in comparison to existing benchmarks.

In [21], a multi-modal ensemble classification approach was investigated for human breast cancer prognosis. The authors proposed a deep learning-based stacked ensemble model using three datasets: CNV, clinical, and gene expression. They proposed a novel two-phase framework where features were extracted using a convolutional neural network in the first phase and RF was implemented in the second phase for output prediction. The results validated the effectiveness of the proposed multi-modal classification. However, there is still a need for a model that can effectively predict breast cancer patient prognosis and survivability. Existing benchmark models only work for a limited number of gene signatures or use similar neural networks for multi-modal data. Therefore, this work aims to design a heterogeneous model for multi-modal data to test the effectiveness of the proposed model in terms of accuracy.

## 3. Methods and Materials

### 3.1. Dataset

The METABRIC dataset is used for this work, extracted from 1980 valid patient records. The METABRIC dataset comprises multi-dimensional data forms such as gene expression, copy number variation, and clinical information for breast cancer [21]. The total number of samples (patient count = 1980) was categorized into two subdivisions: long-term survivors (>five years) and short-term survivors (<5 years). The total number of samples comprises 1489 patient records for long-term survivors and 491 for short-term survivors. The remaining 64 patients of the total samples are alive, but the records of 3.2% of the total sample had incomplete five-year follow-up records. In these cases, we cannot determine whether the patients were long-term survivors or if they died within five years. Therefore, we continue our study by labeling these records as long-term survivors in EBCSP frameworks. This assumption is based on the very high survival chances reported by METABRIC for 64 unpublished patients. The duration of survival for the recorded patients was 125.1 months, whereas the median diagnosis age was 61 years. Using the survival threshold of five years, long-term survivors are labeled as ‘0’ and short-term survivors are labeled as ‘1’ for the binary classification model. The gene-expression (gene exp) and copy number variation (CNV) data have missing values, which are imputed using the weighted nearest neighbor algorithm [22]. The features of gene exp were categorized into three subdivisions according to Gevearat et al. [23]: underline, baseline, and overexpression [−1,0,1]. Underline genes are expressed at a lower level than expected, baseline genes are expressed at the expected level and are used as a control for the comparison of underline and overexpression, and overexpression refers to genes that are expressed at a higher level than expected. These categorized subdivisions help us to understand the state of the cancer and give deep insights into the molecular mechanisms. Clinical data need to be normalized between certain ranges. The min–max algorithm is a simple way of scaling clinical data into a specified range [0,1] by identifying the minimum value $\left[min\left(x_{i}\right)\right]$ and maximum value $\left[max\left(x_{i}\right)\right]$ from the dataset. For each value in the clinical data, subtract the minimum value and divide it by the range $max\left(x_{i}\right)-min\left(x_{i}\right)$. This will scale the clinical data into a range of [0,1]. The clinical data are normalized using the following equation:

(1) $$x_{i,n}^{\prime }=\frac{x_{i,n}-min\left(x_{i}\right)}{max\left(x_{i}\right)-min\left(x_{i}\right)}\left(nMax-nMin\right)+nMin.$$

The overall summary of the dataset is shown in Table 1 and is publicly available at https://github.com/USTC-HIlab/MDNNMD (accessed on 6 October 2021).

### 3.2. Feature Extraction

The well-known problem in sequenced data is the dimensionality curse or high dimensionality, low samples size (HDLSS) for human breast cancer prognosis [24]. Our work focuses on three data modalities: gene exp, CNV, and clinical data, with 24,000, 26,000, and 27 features, respectively. The HDLSS may lead to the worst results for deep learning algorithms [25]. Simultaneously, the success of a learning algorithm is heavily dependent on feature selection when dealing with a large number of features. One popular method for reducing dimensionality is the Minimum Redundancy Maximum Relevance (mRMR) [26,27]. Therefore, mRMR was applied for feature selection from the available data modalities and to reduce the dimensionality without losing significant information. The final selection of features was made by considering the AUC value of a different set of features. An incremental approach was used for feature selection by applying the mRMR algorithm, selecting the top 100 features, and evaluating the model performance. Then, the top 200 and 500 features were validated using the AUC in this way. By evaluating the AUC for the selected set of features, we found N’s best features with a step size of 100. At last, 400 gene exp, 200 CNV, and 25 clinical data features were considered for the EBCSP framework, which can be viewed in Table 2.

## 4. Experimental Setup

**Figure 2:**
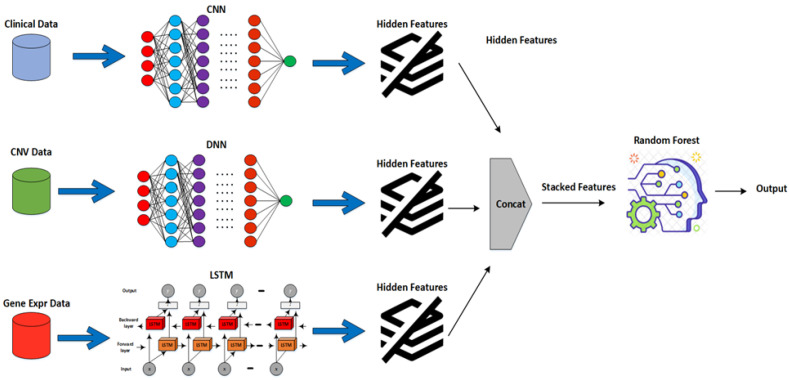
Proposed EBCSP framework working view.

Ten cross-folds were used to evaluate the EBCSP framework in an experimental setup. The available dataset of 1980 samples was randomly divided into 10 subgroups. We merged nine of these subgroups to create the training set, while one subgroup was set aside as the testing set. Additionally, the training set was further divided into an 80% training set and a 20% validation set. Figure 2 provides a visual representation of the entire experimental setup for the EBCSP framework.

### 4.1. DNN, CNN, and LSTM for Individual Modalities

**Figure 3:**
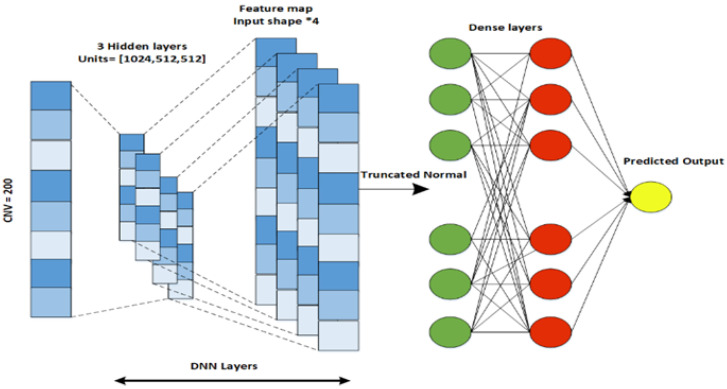
DNN architecture designed for CVN data.

The proposed EBCSP model utilizes a deep neural network, convolutional neural network, and recurrent neural network for predicting human breast cancer prognosis. These models extract features from the individual data modalities to be used in the subsequent phase of the EBCSP model. Specifically, the EBCSP model composed of a DNN, CNN, and RNN-LSTM has been used with multiple learning filters applied directly to the data modalities. Among these models, the CNN is applied to the clinical data, the DNN works on the CNV data, and the RNN-LSTM is designed for the gene expression data modality. A hierarchy of hidden layers was designed for the DNN architecture of the CNV data modality. The combination of lower-level features was used to extract higher-level features. The DNN consists of an input layer, multiple hidden layers, and an output layer, with fully connected units in each layer, as shown in Figure 3. The number of layers (N) is five, including an output layer, and the Tanh activation function is used on the hidden layer to capture the nonlinear relationships within the data. The sigmoid activation function is used in the output layer for efficient binary classification. To prevent overfitting, the dropout regularization technique was applied to each hidden layer [28].

**Figure 4:**
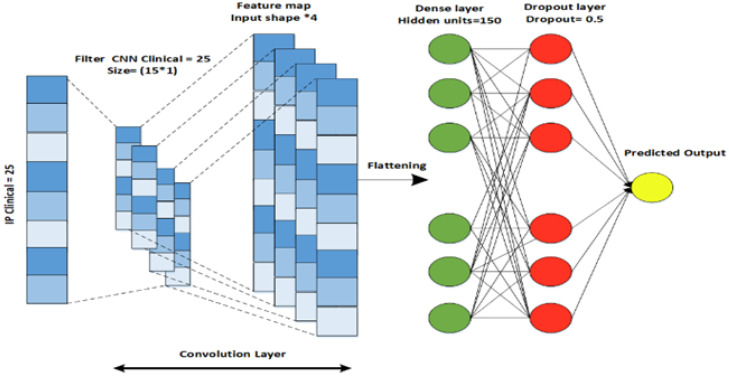
CNN architecture designed for Clinical data.

Similarly, a CNN architecture is designed for the clinical data modality that extracts features from the previous layers for the next layer. A CNN captures the input from the dataset and passes each datum through a convolutional layer of specific filters. As a result of a convolutional process, a feature map is produced. A feature map is an element-wise product followed by the addition of an input matrix between the filter matrix and the corresponding value. The CNN architecture consists of five layers, including an input layer, a convolutional layer, a flattening layer, a dense layer, and an output layer, as shown in Figure 4. To control the feature size, padding is used after the flattening is performed for the output of the convolutional layer. The flattened output is then passed through the fully connected dense layer. L2 regularization is used to prevent the overfitting of the model [29].

**Figure 5:**
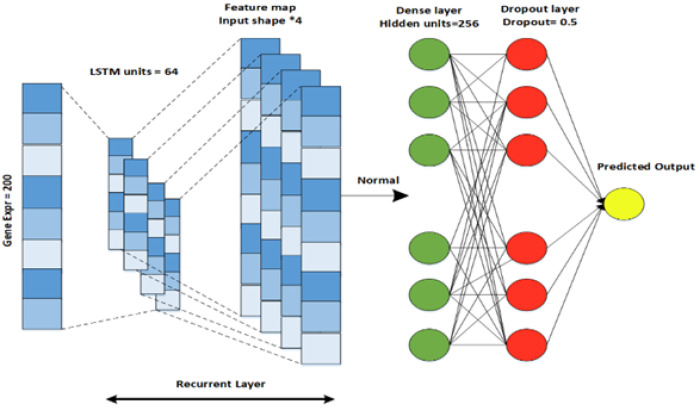
RNN-LSTM architecture designed for Gene Expression data.

For gene expression data, a LSTM (long short-term memory) model is designed. LSTM is an improved version of a recurrent neural network that contains a set of memory blocks [30]. By adding the cell states, LSTM saves that state for a long time. LSTM is used to recall and collaborate the data from the previous layer and the active layer. The model comprises an LSTM layer, a dense layer with a dropout regularization technique, and an output layer that can be seen in Figure 5. The Relu activation function is used in this model because it does not activate all the neurons at a single moment and reduces the negative value to zero. In the above three models, the Glorot normal initializer [31] is used to obtain the initializing filter values. The initializer selects the values whose mean is equal to zero and the standard deviation range is in between $-\sqrt{\frac{2}{n_{i},n_{o}}},\sqrt{\frac{2}{n_{i},n_{o}}}$, where $n_{i}$ and $n_{0}$ are the input and output values. DNN, CNN, and LSTM use the constant seed value 0.1. The Adam optimizer is used for model training. The parallel cross-entropy is used and upgraded with an enhancer, as it is computationally efficient, requires little memory, and is well-suited to large problems with additional data. A loss function is used to measure how well a deep learning classifier fits the empirical data [32]. As our problem is a binary classification problem, the ground truth (y) can only have two states, one or zero. Therefore, a binary cross-entropy loss was applied in this study to assess the prediction performance of the deep learner. The loss function is defined as:

(2) $$L\left({\hat{y}}_{t},{\hat{y}}_{t}\right)=\frac{-1}{N}\sum_{i=0}^{N}\left[y_{t}\left(i\right)logy_{t}\left(i\right)-\left(1-y_{t}\left(i\right)\right)log\left(1-y_{t}\left(i\right)\right)\right]+\frac{1}{2}\pi \sum_{k=1}^{k}\sum_{j=1}^{nk}\sum_{i=1}^{mk}W_{ij}^{k^{2}}$$

In the above equation, *L* represents the cost function, which measures the errors between the actual and the predictive score. $y_{t}\left(i\right)$ and ${\hat{y}}_{t}\left(i\right)$ are the actual label and predictive scores for class i. The detailed parameters of the models are shown in Table 3.

### 4.2. EBCSP Model for Multi-Dimensional Data

The proposed EBCSP model is characterized into two sub-divisions, as explained below.

#### 4.2.1. Feature Extraction and Stacking Phase

Multi-modal datasets have different data representations when combined directly as an input to a deep learning model, and the model may therefore not produce efficient results [33]. Therefore, in the EBCSP framework, the concerned modalities were not combined directly. Instead, a single data modality is passed to the learning model for feature extraction, ensuring heterogeneity between the different models of each data modality. The proposed model uses three types of multi-modal data, namely, clinical, copy number variation, and gene expression data, for breast cancer prognosis using different learning models such as CNN, DNN, and LSTM. In the feature extraction phase, we use neural networks to extract features and use the AUC as the performance measurement criterion. We then combine the extracted features to form stacked features.

#### 4.2.2. Prognosis Phase

The stacked features obtained from the previous phase are passed to the random forest algorithm. According to the literature, various machine learning classifiers have been applied to breast cancer datasets, such as support vector machine, logistic regression, Naive Bayes, and random forest [6,21]. Among them, random forest has been shown to predict breast cancer diagnosis better than other classifiers. The proposed model is generic, so any machine learning classifier can be used for the final binary classification, i.e., short-term and long-term survival. The predicted output from the random forest is the final output for the EBCSP framework. To evaluate the performance of the model, we plot the Receiver Operating Characteristics Curve (ROC) [34] between the false positive (1-specificity) and true positive (sensitivity) rates and compute the AUC. Furthermore, the evaluation metrics for the proposed framework are sensitivity (Sn), specificity (Sp), precision (Pre), and accuracy (Acc) [31] and are defined as follows:

(3) $$Sensitivity=\frac{Tp}{Tp+Fn}$$

(4) $$Specificity=\frac{Tn}{Tn+Fn}$$

(5) $$Precision=\frac{Tp}{Tp+Fp}$$

(6) $$Accuracy=\frac{Tp+Tn}{Tp+Tn+Fp+Fn}$$

In the above equations, $T_{p}$, $F_{p}$, $T_{n}$, and $F_{n}$ indicate true positive, false positive, true negative, and false negative, respectively.

## 5. Results and Discussions

The section below present a detailed comparison of the EBCSP framework with existing benchmarks.

### 5.1. EBCSP Model Performance Evaluation

**Figure 6:**
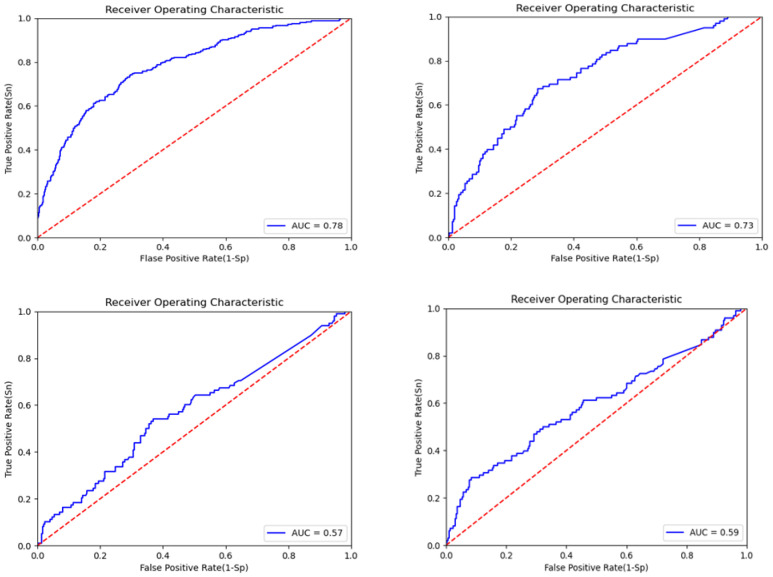
ROC curve for the EBCSP model feature extraction for prognosis prediction.

To confirm the effectiveness of multi-modal data, we employed individual deep learning models for each data modality. The key evaluation parameter for feature extraction from individual models was the AUC plot, along with the accuracy. The model with a higher AUC value was considered to be better than the one with a lower AUC value. The AUC plot gain for CNN-Clinical is 0.78 (epoch = 20); for DNN-CNV, it is 0.73 (epoch = 20); and for LSTM-gene exp, we observe a 0.57 gain with 20 epochs and 0.59 with 32 epochs, as depicted in Figure 6. For the EBCSP model, we used features extracted from 32 epochs. The accuracy gained by the individual models were 78.28%, 74.65%, and 76.26% for CNN-clinical, DNN-CNV, and LSTM-Gene Exp, respectively.

To address the variance problem caused by the limited dataset, we utilized ten cross-folds to evaluate the EBCSP model using the Metabric dataset, which includes 1980 samples. The samples were randomly divided into 10 subgroups; among these, 9 subgroups were taken as training sets and 1 as the validation set. The output of the individual learning models is integrated and considered as stacked features. It is evident from the literature that RF outperforms stacked features as compared to other classifiers [18]. Therefore, we used RF for the stacked features for the final classification and validated the classification with different parameters, where we acquire the confusion metrics, sensitivity, F1 score, precision, specificity, and accuracy of the EBCSP framework.

For the validation set, we have 495 total samples. Among these samples, we have 368 samples accurately predicted as long-term survivors and 121 are genuinely predicted as short-term survivors. There were no false predictions for long-term survivors, but six samples were falsely predicted as short-term survivors. The absence of false positives may be due to imbalanced classes. The discussion above shows that 489 samples were predicted correctly, and 6 samples were falsely predicted; the results are statistically shown in Table 4.

As the proposed model is designed for binary classification, it was evaluated for each class using precision, recall, and F1 score parameters. The results show that the model had scores of 0.98, 1.0, and 0.99 for precision, recall, and F1-score, respectively, for the long-term class, whereas the short-term class model had scores of 1.00, 0.95, and 0.98 for the considered evaluation parameters, as shown Table 5.

The final evaluation of the model is performed after passing the stacked features to the RF, where the model produces the final prediction. The final results from the RF are evaluated based on Sn, Sp, Pre, F1-score, and Acc. The model generates outstanding prediction results with a 1.0 sensitivity rate, 0.95 specificity, 0.98 precision, 0.99 F1-score, and 0.98 accuracy. The overall result metrics are given below in Table 6.

### 5.2. EBCSP Model vs. Existing Benchmarks

**Figure 7:**
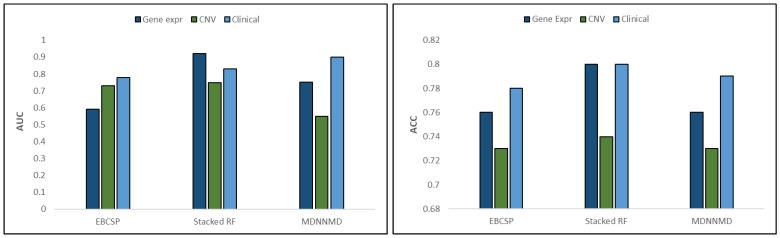
AUC and ACC comparison between data modalities.

The above results indicate that the EBCSP model outperforms existing benchmarks. We compare the proposed EBCSP model with existing models such as multi-modal deep neural networks by integrating multi-dimensional data (MDNNMD) [6] and the Stacked RF-based ensemble model [18]. We compute the AUC for learning models and prediction results. The learning models exhibit AUC scores of 0.60, 0.75, and 0.80 for LSTM gene exp, DNN CNV, and CNN clinical data, respectively. The accuracy measures of the learning models were 0.78, 0.74, and 0.80 for LSTM gene exp, DNN CNV, and CNN clinical data, respectively. The individual training model results were up to the mark compared to the existing benchmarks, as shown in Figure 7.

**Figure 8:**
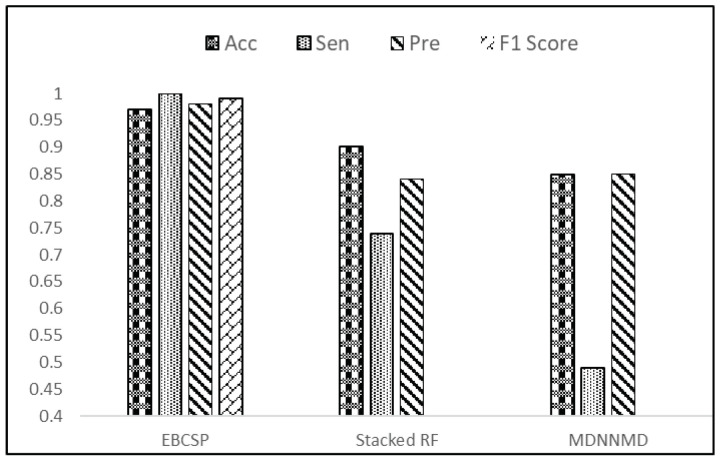
Result evaluation of EBCSP model with existing benchmarks.

The final prediction made by the EBCSP model shows a higher AUC value of 0.97, whereas the Stacked RF and MDNNMD models score AUCs of 0.93 and 0.84, respectively. After the prognosis of breast cancer, telemedicine can be adapted for treatment as it is a safe healthcare method [35,36]. The AUC value ensures the adequate performance of the proposed model. In terms of accuracy, the EBCSP model shows better prediction, with an accuracy score of 0.98%, which is higher than the 0.90% and 0.82% accuracy scores of the Stacked RF and MDNNMD models. The proposed model is also evaluated according to Sn, Sp, Pre, and F1-score, with results of 1.0, 0.95, 0.98, and 0.99, respectively. The EBCSP model shows better results than the existing benchmarks, as shown in Figure 8.

## 6. Conclusions

Breast cancer is a severe illness that often results in poor outcomes and is a leading cause of mortality. Physicians can make informed decisions about patient care by assessing patients’ survival rates. Therefore, there is a pressing need for a rapid and effective computational model to predict human breast cancer prognosis. This study aims to develop a prognostic model for human breast cancer by using independent neural networks for each relevant data modality. The EBCSP model, which stacks multi-dimensional data, is presented in this study for predicting the survival rate (long-term or short-term) of patients with human breast cancer. Clinical, gene expression, and copy number variation data are all informative sources for breast cancer prognosis. Individual neural network models were designed for each data modality, and the outputs were combined using RF for final classification. The predicted output can be validated or integrated with other sources of information before being used for clinical decision making. The EBCSP model outperforms existing benchmarks, including MDNNMD and stacked RF, and can be extended to similar critical diseases. However, it is important to note that the proposed work is limited to additional data modalities such as miRNA and gene methylation data, and future studies will explore these modalities using innovative methodologies.

## Figures and Tables

**Table 1:** METABRIC Dataset Summary.

Renounce # Year	5 Year
Patient Count	1980
Survivors #Long Term	1489
Survivors #Short Term	491
Diagnosis Median Age	61
Average Survival Month	125.1

**Table 2:** Count of selected features.

Data Set	Total Features	Selected Features
CNV	26,298	200
Gene Expression	24,368	400
Clinical	27	25

**Table 3:** Learning parameters of EBCSP framework.

Features	CNN-Clinical	DNN-CNV	LSTM-Gene Exp
Initializer	Adam	Adam	Adam
Initializer constant	0.1	0.1	0.1
Number of layers	1 (convolutional)	4 (hidden)	1 (hidden)
Number of filters	25, 4	-	-
Kernel size	15	64	-
Stride	2	-	-
Padding	Same	-	-
Learning Rate	10-3	10-3	10-3
Number of hidden layers	1	4	1
Hidden units	150	[1024,512,512,512]	256
Dropout	-	0.5	0.5
Training Epoch	20	20	52
Batch size	8	8	8
Activation Function	Tanh	Tanh	Relu
Loss Function	L2 regularization, Binary cross entropy	L2 regularization, Binary Cross entropy	L2 regularization, Binary Cross entropy

**Table 4:** Confusion Metrics for the validation set.

	Long-Term Survivors	Short-Term Survivors
Long-Term Survivors	368	0
Short-Term Survivors	6	121

**Table 5:** Class evaluation of the model.

	Precision	Recall	F1 Score
Long-Term Survivors	0.98	1.00	0.99
Short-Term Survivors	1.00	0.95	0.98

**Table 6:** Overall model performance.

Evaluation Parameters	EBCSP Results
Sensitivity (Sn)	1.00
Specificity (Sp)	0.95
Precision (Pre)	0.98
F1-Score	0.99
Accuracy (Acc)	0.98

## Data Availability

Data are available from the corresponding author upon reasonable request.

## Abbreviations

The following abbreviations are used in this manuscript:

EBCSP	Ensemble model for breast cancer survivability prediction
DNN	Deep Neural Network
CNN	Convolutional Neural Network
RNN	Recurrent Neural Networks
LSTM	Long short-term memory
RF	Random Forest
AECOX	Auto-Encoder with Cox regression networks
